# Purification of nanogram-range immunoprecipitated DNA in ChIP-seq application

**DOI:** 10.1186/s12864-017-4371-5

**Published:** 2017-12-21

**Authors:** Jian Zhong, Zhenqing Ye, Samuel W. Lenz, Chad R. Clark, Adil Bharucha, Gianrico Farrugia, Keith D. Robertson, Zhiguo Zhang, Tamas Ordog, Jeong-Heon Lee

**Affiliations:** 1 Epigenomics Development Laboratory, Epigenomics Program, Center for Individualized Medicine, Mayo Clinic, Rochester, MN 55905 USA; 2 Division of Biomedical Statistics and Informatics, Department of Health Science Research, Mayo Clinic, Rochester, MN 55905 USA; 3 Division of Gastroenterology and Hepatology, Department of Medicine, Mayo Clinic, Rochester, MN 55905 USA; 4 Enteric Neuroscience Program, Mayo Clinic, Rochester, MN 55905 USA; 5 Department of Molecular Pharmacology and Experimental Therapeutics, Mayo Clinic, Rochester, MN 55905 USA; 6 Department of Biochemistry and Molecular Biology, Mayo Clinic, Rochester, MN 55905 USA; 7 Epigenomics Program, Center for Individualized Medicine, Mayo Clinic, Rochester, MN 55905 USA; 8 Department of Pediatrics and Department of Genetics and Development, Institute for Cancer Genetics, Columbia University, New York, NY 10032 USA; 9 Department of Physiology and Biomedical Engineering, Mayo Clinic, Rochester, MN 55905 USA

**Keywords:** Nanogram DNA, ChIP-seq, DNA purification, DNA storage

## Abstract

**Background:**

Chromatin immunoprecipitation-sequencing (ChIP-seq) is a widely used epigenetic approach for investigating genome-wide protein-DNA interactions in cells and tissues. The approach has been relatively well established but several key steps still require further improvement. As a part of the procedure, immnoprecipitated DNA must undergo purification and library preparation for subsequent high-throughput sequencing. Current ChIP protocols typically yield nanogram quantities of immunoprecipitated DNA mainly depending on the target of interest and starting chromatin input amount. However, little information exists on the performance of reagents used for the purification of such minute amounts of immunoprecipitated DNA in ChIP elution buffer and their effects on ChIP-seq data. Here, we compared DNA recovery, library preparation efficiency, and ChIP-seq results obtained with several commercial DNA purification reagents applied to 1 ng ChIP DNA and also investigated the impact of conditions under which ChIP DNA is stored.

**Results:**

We compared DNA recovery of ten commercial DNA purification reagents and phenol/chloroform extraction from 1 to 50 ng of immunopreciptated DNA in ChIP elution buffer. The recovery yield was significantly different with 1 ng of DNA while similar in higher DNA amounts. We also observed that the low nanogram range of purified DNA is prone to loss during storage depending on the type of polypropylene tube used. The immunoprecipitated DNA equivalent to 1 ng of purified DNA was subject to DNA purification and library preparation to evaluate the performance of four better performing purification reagents in ChIP-seq applications. Quantification of library DNAs indicated the selected purification kits have a negligible impact on the efficiency of library preparation. The resulting ChIP-seq data were comparable with the dataset generated by ENCODE consortium and were highly correlated between the data from different purification reagents.

**Conclusions:**

This study provides comparative data on commercial DNA purification reagents applied to nanogram-range immunopreciptated ChIP DNA and evidence for the importance of storage conditions of low nanogram-range purified DNA. We verified consistent high performance of a subset of the tested reagents. These results will facilitate the improvement of ChIP-seq methodology for low-input applications.

**Electronic supplementary material:**

The online version of this article (10.1186/s12864-017-4371-5) contains supplementary material, which is available to authorized users.

## Background

It is clear that the epigenetic dysregulation is deeply involved in the etiology of various human diseases. Chromatin Immunoprecipitation in combination with next-generation sequencing (ChIP-seq) is a highly informative epigenetic approach as it reveals genome-wide distribution profiles of histone marks, transcription factors, and chromatin-associated proteins [1]. The methodology is well established in cultured cells and indeed the vast majority of ChIP-seq data are generated from cell lines. Current ChIP protocols typically require 5–10 million cells per ChIP [2, 3], which limits the use of ChIP-seq technology in primary cells, rare cell populations, and small clinical samples such as needle biopsies. Furthermore, whereas quality control standards for ChIP-seq studies have been established [3], several key steps still require further optimization, particularly in small and patient-derived samples, where high degree of consistency and efficiency must be achieved before the technique is introduced into clinical medicine.

The workflow of a typical ChIP-seq experiment consists of multiple steps including sample collection, chromatin input preparation, immunoprecipitation, purification of immunopreciptated DNA, library preparation, next-generation sequencing, mapping of sequencing reads, and data analysis [3]. In our efforts to improve the technology, we have realized that purification of immunoprecipitated DNA and storage of the purified DNA, two steps that have received little attention, are critical for successful library preparation and overall ChIP-seq quality. The yield of immunoprecipitated ChIP DNA is dependent on several factors including target of interest, starting amount of chromatin input, and antibody used. Typically, ChIP experiment is designed to generate immunoprecipitated DNA in the nanogram range. However, it is often difficult to obtain greater than 1 ng of purified ChIP DNA in some targets such as transcription factors, chromatin-associated proteins, and histone marks with small genomic footprints, and also in some experiments performed in small numbers of cells and patient-derived clinical samples. The PCR amplification-based library preparation method is well accepted in ChIP-seq applications. There are some reports that less than 1 ng of DNA can be used for ChIP-seq library preparation [4]. However, more DNA is typically better and at least 1–10 ng of purified DNA is recommended for consistency and data quality. Currently, there is no report on how purification methods and reagents affect the recovery of nanogram-range immunoprecipitated DNA and how the purified DNA from different reagents impacts on library preparation and ChIP-seq data quality.

In this study, we sought to improve the experimental steps for purification of immunopreciptated DNA and library preparation for ChIP-seq application. The purification yield was tested for nanogram-range immunoprecipitated DNA by ten ready-to-use, commercially available DNA purification reagents and phenol/chloroform extraction. We also showed the potential interference of purification reagent in the downstream application. Logistically, library preparation is usually performed a day or more after purification. We observed that the storage condition is important for the preservation of low nanogram-range purified ChIP DNA. Finally, we selected four better performing reagents in our hands, and tested how these purification reagents impact library preparation and ChIP-seq data quality using 1 ng of immunoprecipitated DNA generated from H3K4me3 or H3K27me3 ChIPs. Our results indicate that the selected purification kits have a minimal effect on the efficiency of library preparation and the resulting ChIP-seq data.

## Results

### DNA recovery is significantly different amongst purification reagents

**Fig. 1:**
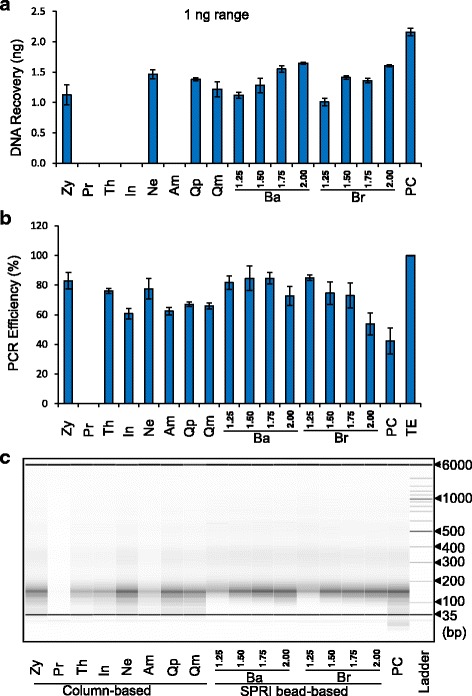
DNA purification reagents vary in their ability to recover low amounts of DNA from de-crosslinked chromatin. **a** Recovered DNA amount by different DNA purification reagents from de-crosslinked chromatin. De-crosslinked chromatin estimated to include 1 ng range DNA in ChIP elution buffer was purified following the manufacturer’s instructions. The data were generated from triplicate DNA samples derived from three independent preparations. Zy, ChIP DNA Clean & Concentrator™ (Zymo Research); Pr, Wizard® SV Gel and PCR Clean-Up System (Promega); Th, GeneJET PCR Purification Kit (Thermo Fisher Scientific); In, PureLink® PCR Purification Kit (Invitrogen); Ne, Monarch® PCR & DNA Cleanup Kit (New England Biolabs); Am, Chromatin IP DNA Purification Kit (Active Motif); Qp, QIAquick PCR Purification Kit (Qiagen); Qm, MinElute PCR Purification Kit (Qiagen); Ba, Agencourt AMPure XP kit (Beckman, chromatin to beads ratio from 1:1.25 to 1:2); Br, RNAClean™ XP kit (Beckman, chromatin to beads ratio from 1:1.25 to 1:2); PC, phenol/chloroform extraction. **b** Interference of PCR amplification by purified eluent of purification reagents. 9 μL eluent was mixed with 1 μL 166 bp of *Drosophila* probe DNA (0.0001 ng), and the resulting mixture was used as the template in 20 μl of real-time PCR reaction. The Ct value for Drosophila probe DNA from TE buffer was set as 100%. The experiment was repeated 3 times using de-crosslinked chromatin estimated to include 1 ng of DNA. **c** Size profiles of DNA purified by different reagents. The DNAs purified from de-crosslinked chromatin estimated to include 50 ng range DNA was analyzed by AATI Fragment Analyzer. DNA size (bp) is shown

The typical ChIP-seq protocol generates chromatin fragments in the 100–300 bp range using sonication or MNase [3, 5]. We prepared chromatin fragments from HeLa cells using the combination of MNase and sonication, and DNA was purified using a Qiagen MinElute column following our standard ChIP protocol as previously described [6]. DNA was quantified and adjusted to 1 ng, 5 ng, 10 ng, and 50 ng in 100 μL of ChIP elution buffer. DNA was then purified by 11 different purification reagents following the manufacturer’s instructions except for the final elution volume (16 μl). Similar experiments were performed using de-crosslinked chromatin estimated to include 1 ng, 5 ng, 10 ng, and 50 ng of DNA. Under our experimental conditions, the performance of individual reagent is highly consistent between de-crosslinked chromatin (Fig. 1a and Additional file 1a) and purified DNA (Additional file 1b). However, the yields of DNA recovery varied considerably among the kits. The Wizard® SV Gel and PCR Clean-Up System (Promega; Pr), the GeneJET PCR Purification Kit (Thermo Fisher Scientific; Th), the PureLink® PCR Purification Kit (Invitrogen; In) and the Chromatin IP DNA Purification Kit (Active Motif; Am) performed poorly with de-crosslinked chromatin. Consistently, these reagents recovered less than 50% of input DNA with purified DNA even when expected DNA amounts were relatively high (10–50 ng). The ChIP DNA Clean & Concentrator™ (Zymo Research; Zy), the Monarch® PCR & DNA Cleanup Kit (New England Biolabs; Ne), the MinElute PCR Purification Kit (Qiagen, Qm), the QIAquick PCR Purification Kit (Qiagen; Qp), the Agencourt AMPure XP kit (Beckman; Ba) and the RNAClean™ XP kit (Beckman; Br), and phenol/chloroform extraction (Invitrogen; PC) performed well with de-crosslinked chromatin. These reagents recovered 78.1% to 95.7% with 10–50 ng of purified DNA, 81.7% to 96.8% with 5 ng of DNA, and 68.1% to 82.9% with 1 ng of DNA except phenol/chloroform extraction with over 100%. We utilized qPCR approach to check the potential interference of each purification reagent in the downstream application. We combined 9 μl of purified DNA eluent with 1 μl of *Drosophila* DNA and used the resulting mixture as the template of PCR reaction to amplify Drosophila-specific DNA in 20 μl of reaction. The amplification efficiency was calculated compared with TE buffer (Fig. 1b). All of regents except Pr and PC showed over 50% amplification efficiency. Importantly, phenol/chloroform extraction showed highest recovery but the resulting purified eluent significantly interfered with the amplification of Drosophila-specific DNA. DNA size analysis of purified DNA from de-crosslinked chromatin showed that SPRI bead-based reagents lose small DNA fragments below 100 bp even in the 1:2 ratio of chromatin to beads compared with the DNAs purified by column-based reagents and phenol/chloroform extraction. As expected, phenol/chloroform extraction recover the DNA fragments less than 35 bp and SPRI bead-based reagents showed the gradual increase of smaller DNA fragments with the increasing concentrations of beads (Fig. 1c).

### Storage conditions affect the preservation of low amounts of purified ChIP DNA

**Fig. 2:**
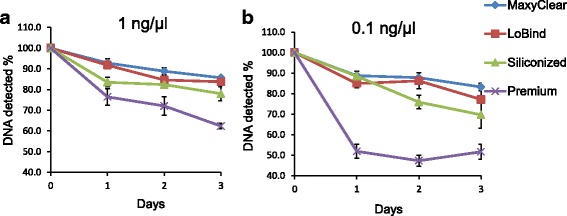
Storage condition of purified ChIP DNA is important. Purified ChIP DNA was adjusted to a concentration of 1 ng/μL (**a**) or 0.1 ng/μL (**b**), aliquoted into 4 different types of microcentrifuge tubes in 15 μL volume, and stored at −20 °C. DNA was quantified using Qubit dsDNA High Sensitivity assay at the indicated time points and expressed as a percentage of the amount measured at day 0. Three independent DNA samples were used in the experiment and DNA concentration from five tubes were measured at each time point. MaxyClear, Axygen® 1.7 mL MaxyClear Snaplock Microcentrifuge Tube; LoBind, Eppendorf DNA LoBind Snap Cap PCR Tube; Siliconized, Fisherbrand™ Siliconized Low-Retention Microcentrifuge Tube; Premium, Fisherbrand™ Premium Microcentrifuge Tube

In collaborations with other laboratories, we observed marked variations in the efficiency of library preparation and ChIP-seq quality among DNA samples from different laboratories in spite of the recovery of similar amounts of purified ChIP DNA following purification. It is common practice to perform library preparation one or more days after ChIP DNA purification. Therefore, we hypothesized that variations in the efficiency of library preparation may in part be caused by the specific conditions under which the purified ChIP DNA is stored, especially the type of the storage tube used. To test this hypothesis, we first chose different kinds of polypropylene tubes from different vendors based on their claimed features such as maximum recovery, low DNA binding, silicon coating, low-retention, etc. (Additional file 2). Purified ChIP DNA from H3K4me3 ChIP experiment performed in HeLa cells was adjusted to 0.1 or 1 ng/μL with TE buffer, and sufficient number of 15 μL aliquot was transferred to different types of polypropylene tubes and stored at 4 °C or −20 °C. DNA amount remaining in solution from one set of tubes was quantified using Qubit dsDNA high sensitivity assay on days 0, 1, 2, 3, and 7. The results indicated a loss of DNA during storage. The degree of loss varied with the DNA concentration, the type of the storage tube, the temperature, and time (Fig. 2). When 1 ng/μL DNA samples were stored at −20 °C, Axygen® 1.7 mL MaxyClear Snaplock Microcentrifuge Tubes (MaxyClear) performed the best, preserving 92.8%, 88.9%, and 85.6% of ChIP-DNA during 1, 2, and 3 days of storage, respectively. Eppendorf DNA LoBind Snap Cap PCR Tubes (LoBind) performed similarly well at −20 °C, preserving 91.7%, 84.6%, and 83.7% of DNA over the same time frame. Fisherbrand™ Siliconized Low-Retention Microcentrifuge Tubes (Siliconized) and Premium Microcentrifuge Tubes (Premium) lost 16.5% and 23.5%, respectively, after 24 h storage at −20 °C, and there was further loss (22% and 37.6%, respectively) after 3 days of storage. When DNA samples were stored at a concentration of 0.1 ng/μL, MaxyClear and LoBind tubes again performed better than the other two tubes, preserving 88.7% and 84.9% of DNA after 1 day of storage and 83.2% and 77.2% after 3 days of storage at −20 °C (Fig. 2b). We found DNA concentrations change little between days 3 and 7 at −20 °C. Purified ChIP DNA stored at 4 °C showed a similar pattern of loss, but the change was generally greater (data not shown). These results indicate that the type of storage tube can have a major impact on the preservation of purified ChIP DNA, and that storing samples at lower DNA concentrations lead to loss of a higher percentage of DNA.

### Highly correlated ChIP-seq data are generated from different purification reagents

**Fig. 3:**
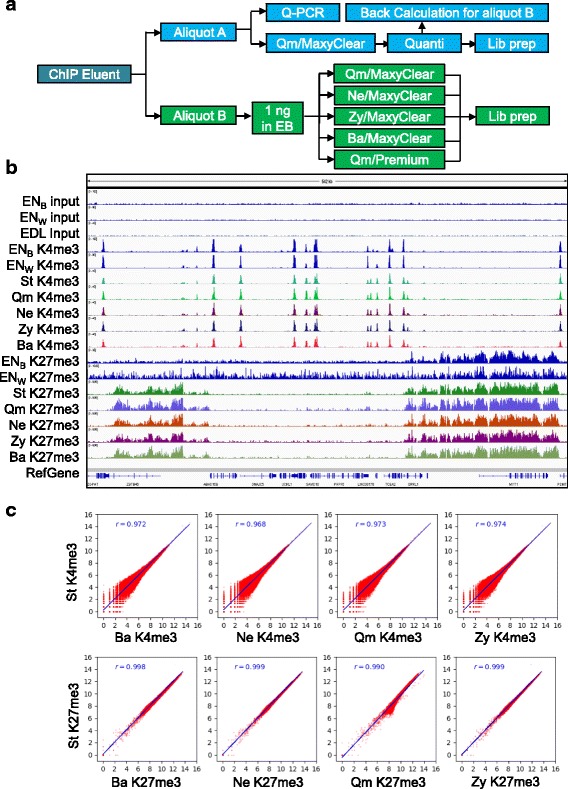
Highly correlated ChIP-seq data are generated from the DNAs purified by different purification reagents. **a** Schematic diagram of the experimental design. **b** Snapshot image of ChIP-seq results generated with different purification reagents as identified in Fig. 1. ChIP-seq results were visualized in a 542 kb genomic region using the Integrative Genomics Viewer [8, 9]. EN_B_ and EN_W_ indicate the ENCODE dataset from Broad Institute and University of Washington, respectively. St, data generated by our standard ChIP-seq protocol. **c** Scatter plots showing the correlation between the internal reference ChIP-seq data obtained with standard protocol for the lab (St) and the corresponding datasets generated with different purification reagents. The genome was divided into bins of 5 kb for H3K4me3 and 100 kb for H3K27me3, and the number of mapped reads in the individual bins was calculated. r, Pearson correlation coefficient. Top and bottom panels show H3K4me3 and H3K27me3 ChIP-seq data, respectively

To investigate whether ChIP-seq data quality differs among the libraries prepared through different purification reagents, we performed ChIP with 4 million HeLa cells per reaction using anti-H3K4me3 and anti-H3K27me3 antibodies by our standard methods [6]. After RNase A and Proteinase K treatment of immunoprecipitated chromatin-DNA complexes, the immunoprecipitated DNA in the ChIP elution buffer was divided into two aliquots (Fig. 3a). Aliquot A was purified by the lab standard protocol using MinElute PCR Purification Kit in a MaxyClear tube. The ChIP enrichment was analyzed by quantitative real-time PCR in positive and negative genomic loci (Additional file 3). One nanogram of this sample was subjected to library preparation and sequencing according to our standard protocol (Fig. 3a and b; St) to obtain internal reference ChIP-seq data. Aliquot B was further divided into aliquots containing DNA equivalent to 1 ng of purified DNA. These aliquots were diluted in 100 μL ChIP elution buffer and purified to MaxyClear tubes using one of four DNA purification kits we previously found to have superior performance from 4 providers based on yield and potential interference with downstream application: Zy, Ne, Qm, and Ba. One sample was purified to a Premium tube by MinElute PCR purification kit: Qm/Premium. Library preparation (including PCR amplification) and sequencing were performed as in the case of aliquot A. The yield and size of library DNA was determined before purification of library DNA. Consistent with the results shown in Fig. 2, the yield of library DNA is low from Premium tube compared with MaxyClear tubes (Please compare the lanes QmM and QmP) (Additional file 4). A similar yield of library DNAs was observed among DNAs purified by different purification reagents (data not shown).

The mapping and global enrichment results from the ChIP-seq experiments are shown in (Additional files 5 and 6) [3, 7]. Mapping rates, library complexity, and peak numbers were similar among the libraries prepared with different purification reagents (Additional files 7 and 8). Peak profiles visualized in the Integrative Genomics Viewer (IGV; Broad Intitute, Cambridge, MA) [8, 9] were also highly similar among the datasets including the results generated using our standard protocol (St) (Fig. 3b). As expected [10, 11], narrow H3K4me3 peaks are primarily found at active promoters (see genes in the middle), whereas broad H3K27me3 peaks are distributed over PRC2-repressed genes such as MYT1 and ZBTB46. The H3K4me3 peak profiles also closely matched corresponding data generated by the ENCODE consortium in HeLa cells [12]. Although similarities could also be found between our H3K27me3 data and the corresponding ENCODE results, the data from University of Washington were considerably less clear due likely to suboptimal enrichment.

To formally analyze the correspondence among the data generated with the different purification reagents, we performed Pearson correlation analysis between H3K4me3 and H3K27me3 ChIP-seq dataset generated with our standard protocol and each of the datasets obtained using the tested DNA purification kits (Fig. 3c). We observed uniformly high correlation for both marks with Pearson correlation coefficients ranging between 0.990 and 0.999 with *p* < 0.001. Our experimental datasets were also highly correlated with the corresponding ENCODE data (Additional file 9). These results indicate high degree of similarity among ChIP-seq dataset obtained using different DNA purification reagents.

## Discussion

To our knowledge, this is the first report to systematically test the efficacy of purification and library preparation of nanogram-range immunoprecitated DNA in ChIP-seq application. These aspects of ChIP-seq experimentation have received relatively little attention [3], although they may affect success rates and reliability of these demanding experiments. We showed that DNA purification reagents have variable impacts on the recovery of nanogram-range immunoprecipitated DNA in ChIP elution buffer. We also observed the storage condition of purified DNA is important. However, DNA purification reagents have a minimal impact on ChIP-seq data if sufficient amount of DNA is available for library preparation. It is noteworthy that current library preparation technology supports consistent and robust library preparation from over 1 ng of purified DNA. Several groups have reported the development of ChIP-seq protocols for low cell number, expecting to generate from nanogram to picogram range of immunoprecipitated DNA [2, 13, 14]. Further optimization of these and other key steps may help achieve the consistency and efficiency of ChIP-seq experimentation required for its introduction into clinical applications.

Our results have revealed significant differences among DNA purification kits in their ability to recover various low amounts of DNA. Our study was not meant to be comprehensive as many other kits were not included. However, we found that four of the eleven tested reagents were capable of handling low nanogram DNA in ChIP elution buffer and had no noticeable negative impact on library preparation and ChIP-seq data quality (Figs. 1 and 3). Well-performing reagents included both more traditional, silica-membrane-based column purification kits and solid-phase reversible immobilization (SPRI)-based reagents, which utilize paramagnetic beads and can be easily automated. DNA fragments less than 30 bp in size are preferentially lost during column-based purification; whereas SPRI bead-based purification results in loss of DNA less than 100 bp in size (Fig. 1c). Consistently, 1% of sequencing reads from the Agencourt AMPure XP kit is smaller than 100 bp but nearly 5–10% of sequencing reads are smaller than 100 bp from column-based purification reagents (Additional file 10). We tested whether purification reagents utilizing silica columns or SPRI may introduce any bias in ChIP-seq data. The correlation analysis of ChIP-seq data clearly indicated no detectable differences between reagents based on these two principles of operation (Fig. 3; Additional file 9). We also compared the distribution of ChIP-seq peaks from small fragment in column-based purification reagents with the distribution of ChIP-seq peaks from the Agencourt AMPure XP kit. We did not observe any noticeable differences (Data not shown). These results indicate that the differential loss of small fragments do not introduce the bias in ChIP-seq application. However, it is noteworthy to mention that it may be important in other applications involved with DNA fragments smaller than 100 bp.

The lengthy and complicated nature of standard ChIP-seq protocols makes sample storage almost unavoidable e.g. before library preparation. Therefore, we have also tested the effect of key storage conditions on ChIP DNA loss. In these experiments we confirmed that depending on the DNA concentration (0.1–1 ng/μL in 15 μL volume), the duration and temperature of storage, as well as on the tubes used, 7% to >50% of the ChIP DNA could be lost during storage (Fig. 2). In our experiments, the type of polypropylene storage tubes had the greatest impact. The loss of ChIP DNA was greater at 0.1 ng/μL concentration and at 4 °C vs. at −20 °C. Most loss occurred during the first 3 days of storage. These observations are consistent with previous reports of preferential loss of short DNA fragments stored at low concentrations from adsorption to the wall of tubes and denaturation [15–17]. Thus, low amounts of purified ChIP DNA should be stored in low-binding tubes, at −20 °C, and at the highest possible concentration for the shortest possible time. If storage is unavoidable, it is advisable to re-quantify the DNA before library preparation.

## Conclusions

We compared the performance of ten commercial DNA purification reagents and phenol/chloroform extraction on low nanogram quantities of ChIP DNA. Four of the well-performing reagents were selected for investigating the impact on library preparation and ChIP-seq data quality. The selected purification reagents had minimal impact on library preparation and generated highly correlated ChIP-seq data. We also showed that considering storage conditions such as the type of tubes used, DNA concentration, temperature, and duration is critical for maximizing the preservation of low amounts of purified ChIP DNA. Our results will aid efforts directed at the optimization of ChIP-seq methodology for low-input applications including the analysis of small and non-renewable patient samples.

## Methods

### Cell culture and reagents

HeLa cells were purchased from ATCC. Cells were grown in Advanced DMEM (Dulbecco’s Modified Eagle Medium) containing 10% calf bovine serum at 37 °C and 5% CO_2_ with saturating humidity.

### Chromatin immunoprecipitation

HeLa Cells were cross-linked with 1% formaldehyde for 10 min, followed by quenching with 125 mM glycine for 5 min at room temperature. Fixed cells were washed twice with TBS, resuspended in cell lysis buffer (10 mM Tris-HCl, pH 7.5, 10 mM NaCl, 0.5% NP-40), and incubated on ice for 10 min. The lysates were washed with MNase digestion buffer (20 mM Tris-HCl, pH 7.5, 15 mM NaCl, 60 mM KCl, 1 mM CaCl_2_) and incubated for 20 min at 37 °C in the presence of MNase (2000 gel units/4× 10^6^ cells, New England Biolabs, Ipswich, MA). After adding the same volume of sonication buffer (100 mM Tris-HCl, pH 8.1, 20 mM EDTA, 200 mM NaCl, 2% Triton X-100, 0.2% sodium deoxycholate), the lysate was sonicated for 15 cycles (30 s on, 30 s off) using a Diagenode Bioruptor and centrifuged at 15,000 rpm for 10 min. The cleared supernatant equivalent to 4 × 10^6^ cells was incubated with 2 μg of antibody at 4 °C on a rocker overnight. The anti-H3K27me3 antibody (Cat. #9733, Lot #8) was purchased from Cell Signaling Technology (Danvers, MA) and the purified anti-H3K4me3 antibody was generated in-house (EDL Lot 1). After adding 30 μl of prewashed protein G-magnetic beads, the reaction was further incubated for 3 h. The beads were extensively washed with ChIP buffer, high salt buffer, LiCl_2_ buffer, and TE buffer. All washes were carried out for 5 mins at 4 °C on a rotating wheel. Bound chromatin was eluted in 100 μL ChIP elution buffer (10 mM Tris-HCl, pH 8.0, 10 mM EDTA, 150 mM NaCl, 5 mM DTT, 1% SDS) and reverse-crosslinked at 65 °C overnight. After treatment with RNase A and proteinase K, DNA was purified by Qiagen MinElute PCR Purification Kit (Cat. # 28006, Valencia, CA) and quantified using Qubit dsDNA High Sensitivity assay (Invitrogen, Q32851). To check the size of input chromatin, purified input DNA was analyzed by Fragment Analyzer (Advanced Analytical Technologies; AATI; Ankeny, IA) using the High Sensitivity NGS Fragment Analysis Kit (Cat. # DNF-486).

### Analysis of DNA recovery in ChIP elution buffer using different purification reagents

Chromatin input was prepared from HeLa cells following ChIP protocol as described above and was reverse-crosslinked at 65 °C overnight. DNA was purified using MinElute PCR Purification Kit after treatment of RNase A and proteinase K. DNA was quantified using Qubit dsDNA High Sensitivity assay and adjusted to 1 ng/μL with TE buffer. DNAs were prepared to final 1 ng, 5 ng, 10 ng and 50 ng in 100 μL ChIP elution buffer and were purified by 11 different purification reagents as suggested by the manufacturer except for the elution volume, which was fixed at 16 μL. Similarly, DNAs were purified from de-crosslinked chromatin estimated to include 1 ng, 5 ng, 10 ng, and 50 ng of DNA after treatment of RNase A and proteinase K. The following reagents were used in the experiment: ChIP DNA Clean & Concentrator™ (Cat. # D5205) from Zymo Research (Zy) (Irvine, CA); Wizard® SV Gel and PCR Clean-Up System (Cat. # A9281) from Promega (Pr) (Fitchburg, WI); GeneJET PCR Purification Kit (Cat. # K0701) from Thermo Fisher Scientific (Th) (Waltham, MA); PureLink® PCR Purification Kit (Cat. # K310001) from Invitrogen (In) (Carlsbad, CA); Monarch® PCR & DNA Cleanup Kit (Cat. # T1030S) from New England Biolabs (Ne) (Ipswich, MA); Chromatin IP DNA Purification Kit (Cat. # 58002) from Active Motif (Am) (Carlsbad, CA); QIAquick PCR Purification Kit (Cat. # 28106) from Qiagen (Qp) (Valencia, CA), MinElute PCR Purification Kit (Cat. # 28006) from Qiagen (Qm); Agencourt AMPure XP (Cat. # A63881) from Beckman (Ba) (Indianapolis, IN), RNAClean™ XP (Cat. # A63987) from Beckman (Br), and phenol/chloroform extraction (PC) (Additional file 11). The sample-to-beads ratio tested for Ba and Br were 1:1.25, 1:1.50, 1:1.75, and 1:2. Each purification reagent was tested in triplicate DNA samples derived from 3 independent experiments. The recovery rate was calculated by dividing the recovered DNA amount after purification by the starting amount and expressed in percentages. DNA size of purified DNA from de-crosslinked chromatin was analyzed by AATI Fragment Analyzer using the High Sensitivity NGS Fragment Analysis Kit.

### PCR analysis of final eluent from different purification reagents

To check the potential interference of purification reagent in the downstream application, qPCR assay was performed. 9 μl of final DNA eluent from each purification reagent was combined with 1 μl of 166 bp fragment of *Drosophila* probe DNA, and the resulting mixture was used as the template to amplify *Drosophila*-specific probe DNA in 20 μl of real-time PCR reaction. TE buffer was used as control and the Ct value from TE buffer was set as 100%. The experiment was repeated 3 times using the final eluents from de-crosslinked chromatin estimated to include 1 ng of DNA. The following primer sequences were used for *Drosophila* probe preparation and real-time PCR: *Dro*sophila probe-F: 5′- GCTGACGGGAACAATGGTC-3′, *Drosophila* probe-R: 5’-TGGCGACGACGTAACAACAT-3′.

### Analysis of storage conditions for purified ChIP DNA

ChIP DNA was prepared in HeLa cells using H3K4me3 antibody and the protocol described above. Purified ChIP DNA was adjusted to 0.1 or 1 ng/μL with TE buffer. Sufficient number of aliquots were made into 1.5 mL polypropylene-based tubes used in the typical molecular biology laboratory in 15 μL volume, and stored at 4 °C and −20 °C. The following tubes were tested: Axygen® 1.7 mL MaxyClear Snaplock Microcentrifuge Tube (Cat. # MCT-175-C) (Corning, New York), Eppendorf DNA LoBind Snap Cap PCR Tube (Cat. # 022431021) (Hauppauge, NY), Fisherbrand™ Siliconized Low-Retention Microcentrifuge Tube (Cat. # 02–681-331) (Waltham, MA), and Fisherbrand™ Premium Microcentrifuge Tube (Cat. # 05–408-129). DNA was quantified using Qubit dsDNA High Sensitivity assay on days 0, 1, 2, 3, and 7 of storage. At each time point, the DNA amount from 5 individual tubes was measured and the tubes were discarded. The experiment was repeated in triplicate in independently prepared H3K4me3 ChIP DNA samples. DNA amounts detected in solution were compared to the starting DNA amount.

### Library preparation of DNA purified by selected purification reagents and sequencing

ChIP was performed in HeLa cells as described above for H3K4me3 and H3K27me3 marks. After RNase A and proteinase K treatments, immunopreciptated DNA in the ChIP elution buffer was evenly divided into aliquot A and aliquot B. Aliquot A was purified by MinElute PCR Purification Kit, which is the standard protocol in the Epigenomics Development Lab (EDL). The enrichment was analyzed by real-time PCR targeting positive and negative control genomic loci and DNA was quantified using Qubit dsDNA High Sensitivity assay. The DNA concentration in aliquot B was back-calculated from aliquot A. For aliquot B, the immunoprecipitate equivalent to 1 ng of purified ChIP DNA was diluted to 100 μL of ChIP elution buffer. DNA was purified using selected purification reagents according to the manufacturer’s instructions. For the MinElute PCR Purification Kit, DNA was eluted in Maxyclear and Premium tubes. And for other reagents, DNA was eluted in Maxyclear tubes. ChIP-seq libraries were prepared from DNA purified from the aliquot B by the various reagents using the ThruPLEX® DNA-seq Kit V2 (Rubicon Genomics, Ann Arbor, MI) according to the manufacturer’s instructions. For comparison, the libraries were also prepared from 1 ng of purified input and ChIP DNA from aliquot A (EDL standard protocol). Following repair and adaptor ligation steps, the adaptor-ligated DNA was amplified 12 cycles by PCR in 50 μL reaction volume. To analyze the size and quantity of the library DNA, 2 μL of the PCR reaction was analyzed by the AATI Fragment Analyzer using the High Sensitivity NGS Fragment Analysis Kit. The remaining PCR reaction was further purified for sequencing. A total of 11 ChIP-seq libraries were sequenced to 51 base pairs from both ends in the same lane using the Illumina HiSeq 4000 instrument at the Mayo Clinic Medical Genome Facility Sequencing Core.

### Real-time PCR analysis

Real-time PCR analysis was performed using SYBR Green universal PCR mixes (Bio-Rad). The following primer sequences were used in the experiments: H3K4me3-positive control locus: hGAPDH-F: 5’-CCCACTCCTCCACCTTTGAC-3′, hGAPDH-R: 5’-CCCAGCCACATACCAGGAAA-3′. H3K27me3-positive control locus: hMYT1-F: 5’-CCTGCCGTGTGCTGTTTTT-3′, hMYT1-R: 5’-CACAACATGTCCCCTGGAATC-3′. H3K4me3- and H3K27me3-negative control locus: hCh19-intergenic-F: 5’-AGCTTGTCTTTCCCAAGTTTACTC-3′, hCh19-intergenic-R: 5’-TAGCTGTCGCACTTCAGAGGA-3′.

### Mapping and data analysis

Raw sequencing reads were processed and analyzed using the HiChIP pipeline [7] to obtain visualization files and a list of peaks. Briefly, paired-end reads were mapped to the human reference genome (release hg19/GRCh37) by BWA [18] with default settings, and only uniquely mapped reads were used for further analysis. Peaks were called using the MACS2 algorithm [19] for H3K4me3 and SICER [20] for H3K27me3 at FDR < =1%. Fragment size was calculated from properly mapped read pairs. H3K4me3 and H3K27me3 ChIP-seq datasets (Broad institute and University of Washington) generated by the ENCODE consortium [12] in HeLa cells were downloaded from the Gene Expression Omnibus. Correlation analysis was performed by our in-house scripts. All datasets were randomly downsized to 25 million reads. In brief, the whole genome was divided into 5 kb bins for H3K4me3 and 100 kb bins for H3K27me3, and the number of mapped reads in the bin was calculated. The counts by logarithm log_2_(count +1) were used for pairwise correlation analysis with Pearson coefficient. Here, 1 is a pseudo-count to avoid an undefined error of logarithm of zero. The score of FRiP (fraction of reads in peaks) for each sample was calculated by following the method described in [3]. The library complexity was calculated by the Preseq package [21].

## Additional files

Additional file 1: DNA purification reagents vary in their ability to recover low amounts of DNA. **a** Recovered DNA amount by different DNA purification reagents from de-crosslinked chromatin. De-crosslinked chromatin estimated to include 5, 10, 50 ng range of DNA in ChIP elution buffer was purified following the manufacturer’s instructions as described in Fig. 1a. The data were generated from triplicate DNA samples derived from three independent preparations. **b** Recovery rates of nanogram-range DNA by different DNA purification reagents. DNAs were adjusted to final 1 ng, 5 ng, 10 ng and 50 ng in 100 μL ChIP elution buffer and were purified by purification reagents following the manufacturer’s instructions. Percent recovery was calculated from DNA amounts before and after purification. The data were generated from triplicate DNA samples derived from three independent preparations. (PDF 105 kb)

Additional file 2: Microcentrifuge tubes tested in this study. (PDF 88 kb)

Additional file 3: ChIP enrichment analyzed by qPCR. The ChIP DNA was analyzed by qPCR in a region of constitutively active chromatin region (GAPDH-TSS, H3K4me3-positive), a developmentally repressed region (MYT1-TSS, H3K27me3-positive), and an intergenic region (C19, H3K4me3- and H3K27me3-negative) from HeLa cells. Enrichment in the tested loci is shown as the percentage of input. (PDF 87 kb)

Additional file 4: DNA profiles in H3K4me3 and H3K27me3 ChIP-seq libraries generated through different purification reagents. After library amplification with 12 cycles of PCR, DNAs were analyzed by the Fragment Analyzer. Lane 1 (EDL St), the library from 1 ng of purified ChIP DNA from aliquot A; Lane 2 (1 ng QmM), the library from stored DNA in MaxyClear tube after purification by MinElute PCR Purification Kit from aliquot B; Lane 3 (1 ng QmP), the library from stored DNA in Premium Tube after purification by MinElute PCR Purification Kit from aliquot B; Lane 4 (Input), the library from 1 ng of purified input DNA from the aliquot A. Left panel is the profile of H3K4me3 libraries, and right panel is the profile of H3K27me3 libraries. (PDF 123 kb)

Additional file 5: Mapping results of ChIP-seq reads generated through different purification reagents. (PDF 94 kb)

Additional file 6: Measuring global ChIP enrichment by FRiP (fraction of reads in peaks). The score of FRiP (fraction of reads in peaks) was calculated by the previously published method [3]. FRiP scores for H3K4me3 and H3K27me3 ChIP-seq are visualized from the ENCODE reference (EN_B_ and ENw) and the corresponding datasets obtained either with standard protocol for the lab (St) or by using different purification reagents as identified in Fig. 1. (PDF 94 kb)

Additional file 7: Analysis of library complexity by Preseq package. The library complexity was estimated by Preseq package [8] for H3K4me3 and H3K27me3 ChIP-seq data from the ENCODE reference (EN_B_ and ENw) and the corresponding datasets obtained either with standard protocol for the lab (St) or by using different purification reagents as identified in Fig. 1. (PDF 114 kb)

Additional file 8: Overlapping percentage of peaks relative to ENCODE data. (PDF 91 kb)

Additional file 9: ChIP-seq data generated with different purification reagents are highly correlated with the corresponding ENCODE datasets. Scatter plots showing the correlation between the ENCODE reference ChIP-seq data (EN_B_ and ENw) and the corresponding datasets obtained either with standard protocol for the lab (St) or by using different purification reagents as identified in Fig. 1. The genome was divided into 5 kb bins for H3K4me3 and 100 kb bins for H3K27me3, and the number of mapped reads in the individual bins was calculated. r, Pearson correlation coefficient. *P* value in all correlation analysis was 0.001. A and B panels show H3K4me3 and H3K27me3 ChIP-seq data, respectively. (PDF 248 kb)

Additional file 10: Size distribution of sequencing reads from H3K4me3 ChIP-seq data. (PDF 203 kb)

Additional file 11: Purification regents tested in this study. (PDF 153 kb)

## Abbreviations

ChIP: Chromatin immunoprecipitation
H3K27me3: Histone H3 Lysine 27 trimethylation
H3K4me3: Histone H3 Lysine 4 trimethylation
NGS: Next generation sequencing
