# Supplementary figures and images for: Purification of nanogram-range immunoprecipitated DNA in ChIP-seq application
Source: BMC Genomics. 2017 Dec 21;18:985. doi: 10.1186/s12864-017-4371-5 (PMC5740926; doi:10.1186/s12864-017-4371-5)

A

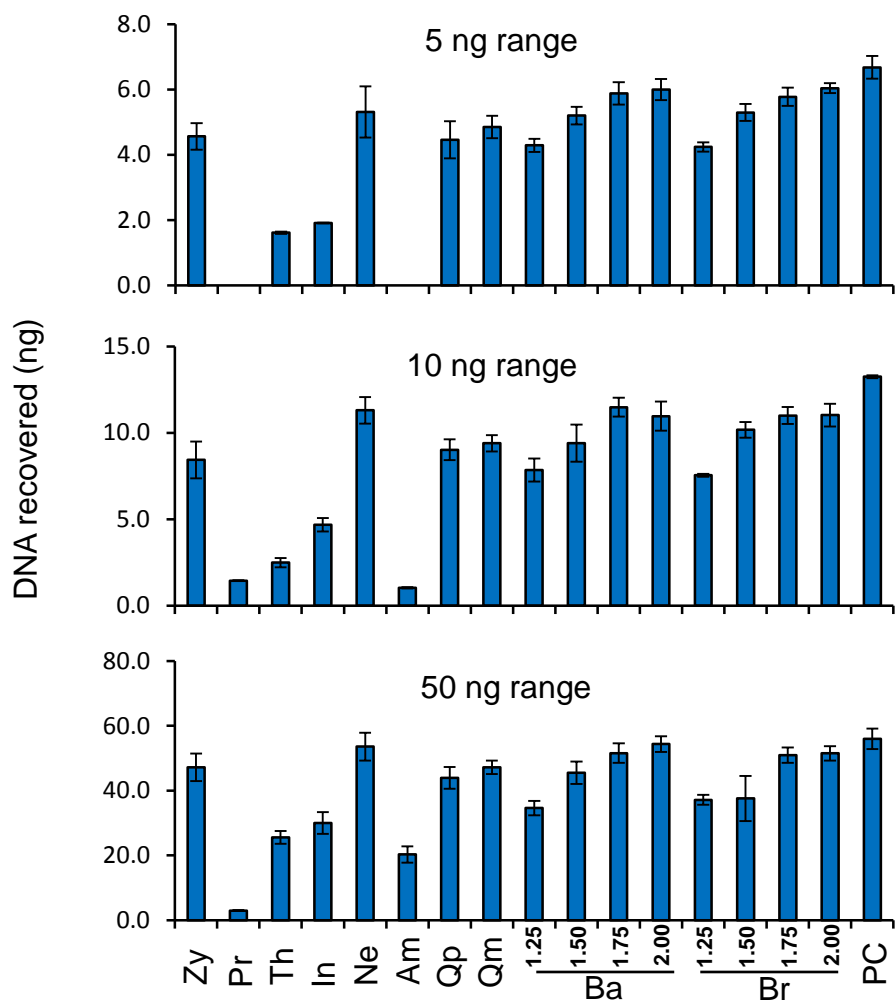

B

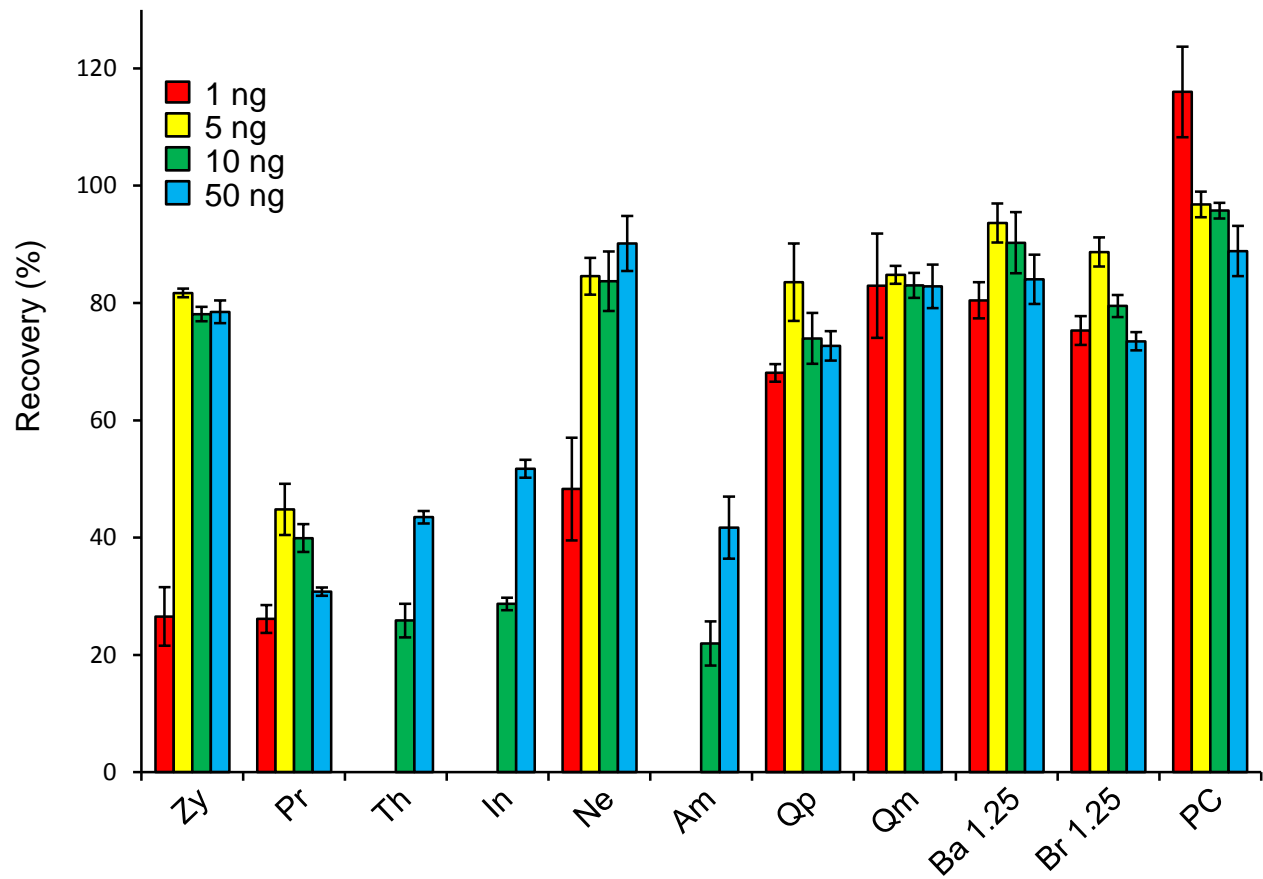

Supplement: Supplementary file 1 — DNA purification reagents vary in their ability to recover low amounts of DNA. a Recovered DNA amount by different DNA purification reagents from de-crosslinked chromatin. De-crosslinked chromatin estimated to include 5, 10, 50 ng range of DNA in ChIP elution buffer was purified following the manufacturer’s instructions as described in Fig. 1a. The data were generated from triplicate DNA samples derived from three independent preparations. b Recovery rates of nanogram-range DNA by different DNA purification reagents. DNAs were adjusted to final 1 ng, 5 ng, 10 ng and 50 ng in 100 μL ChIP elution buffer and were purified by purification reagents following the manufacturer’s instructions. Percent recovery was calculated from DNA amounts before and after purification. The data were generated from triplicate DNA samples derived from three independent preparations. (PDF 105 kb) [file 12864_2017_4371_MOESM1_ESM.pdf]

Additional file 3

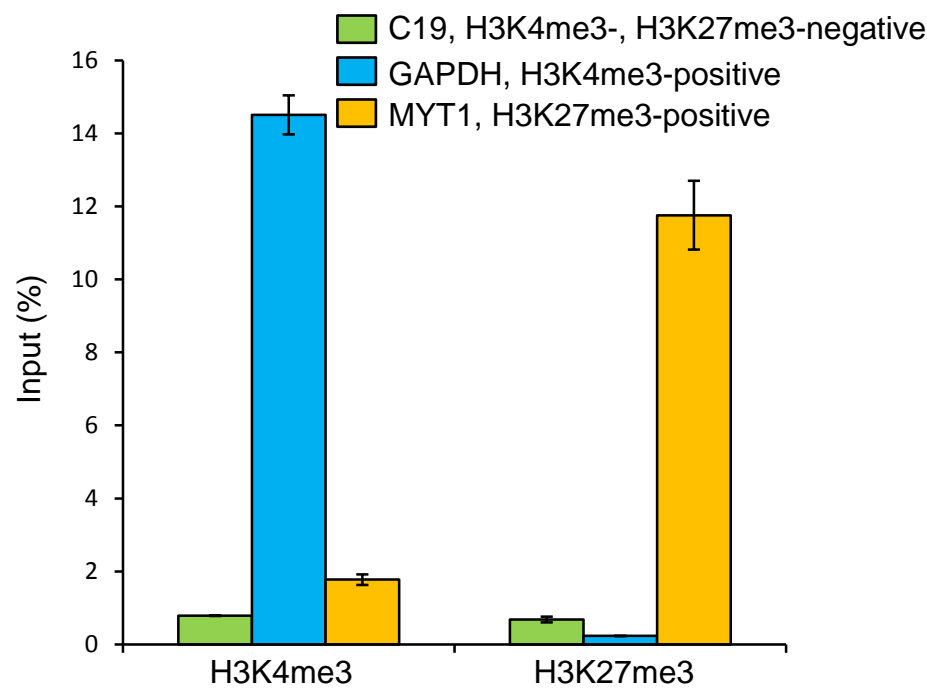

Supplement: Supplementary file 3 — ChIP enrichment analyzed by qPCR. The ChIP DNA was analyzed by qPCR in a region of constitutively active chromatin region (GAPDH-TSS, H3K4me3-positive), a developmentally repressed region (MYT1-TSS, H3K27me3-positive), and an intergenic region (C19, H3K4me3- and H3K27me3-negative) from HeLa cells. Enrichment in the tested loci is shown as the percentage of input. (PDF 87 kb) [file 12864_2017_4371_MOESM3_ESM.pdf]

Additional file 4

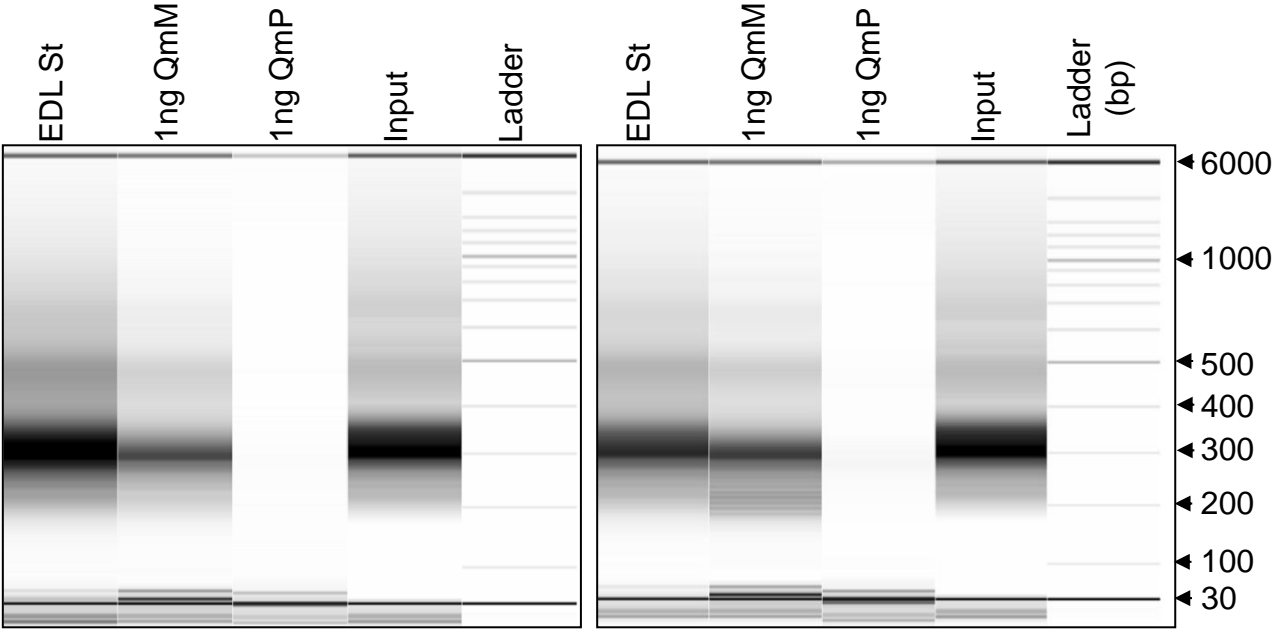

Supplement: Supplementary file 4 — DNA profiles in H3K4me3 and H3K27me3 ChIP-seq libraries generated through different purification reagents. After library amplification with 12 cycles of PCR, DNAs were analyzed by the Fragment Analyzer. Lane 1 (EDL St), the library from 1 ng of purified ChIP DNA from aliquot A; Lane 2 (1 ng QmM), the library from stored DNA in MaxyClear tube after purification by MinElute PCR Purification Kit from aliquot B; Lane 3 (1 ng QmP), the library from stored DNA in Premium Tube after purification by MinElute PCR Purification Kit from aliquot B; Lane 4 (Input), the library from 1 ng of purified input DNA from the aliquot A. Left panel is the profile of H3K4me3 libraries, and right panel is the profile of H3K27me3 libraries. (PDF 123 kb) [file 12864_2017_4371_MOESM4_ESM.pdf]

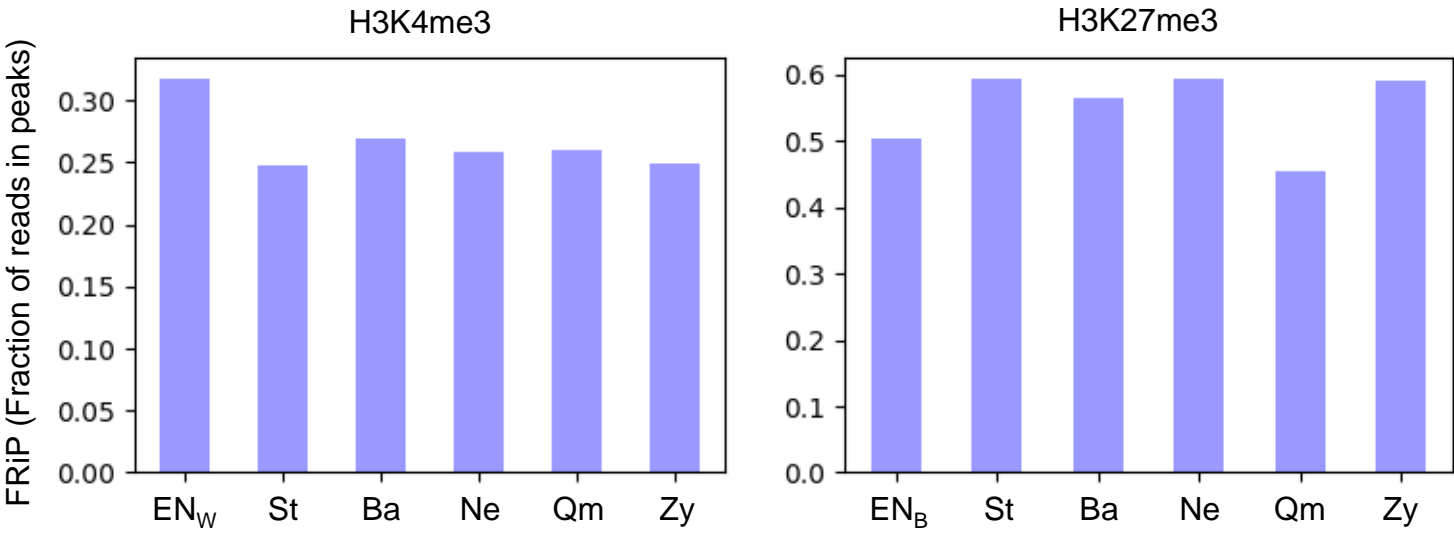

Supplement: Supplementary file 6 — Measuring global ChIP enrichment by FRiP (fraction of reads in peaks). The score of FRiP (fraction of reads in peaks) was calculated by the previously published method [3]. FRiP scores for H3K4me3 and H3K27me3 ChIP-seq are visualized from the ENCODE reference (ENB and ENw) and the corresponding datasets obtained either with standard protocol for the lab (St) or by using different purification reagents as identified in Fig. 1. (PDF 94 kb) [file 12864_2017_4371_MOESM6_ESM.pdf]

A

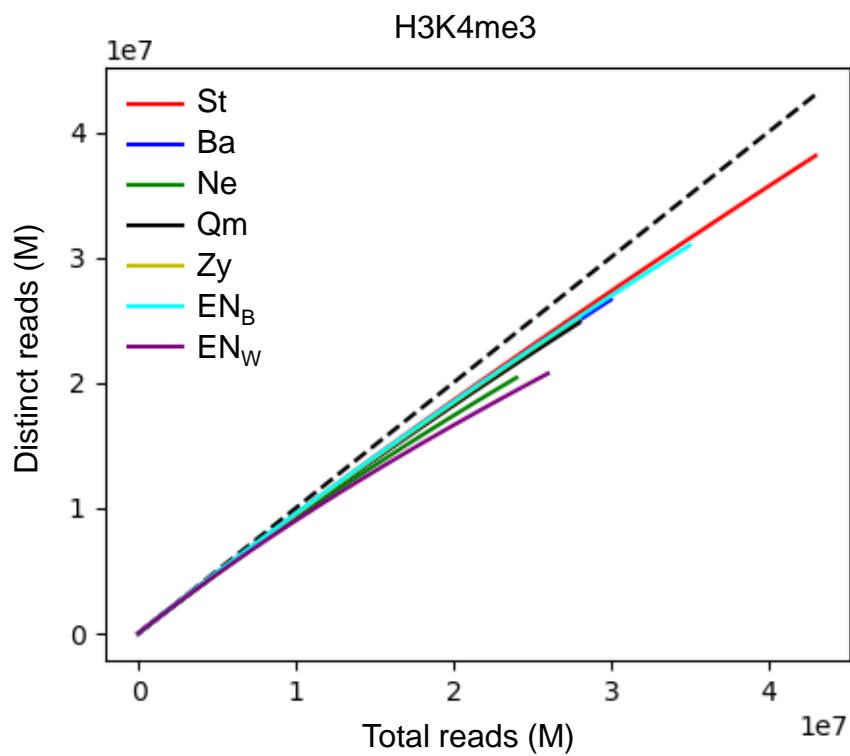

B

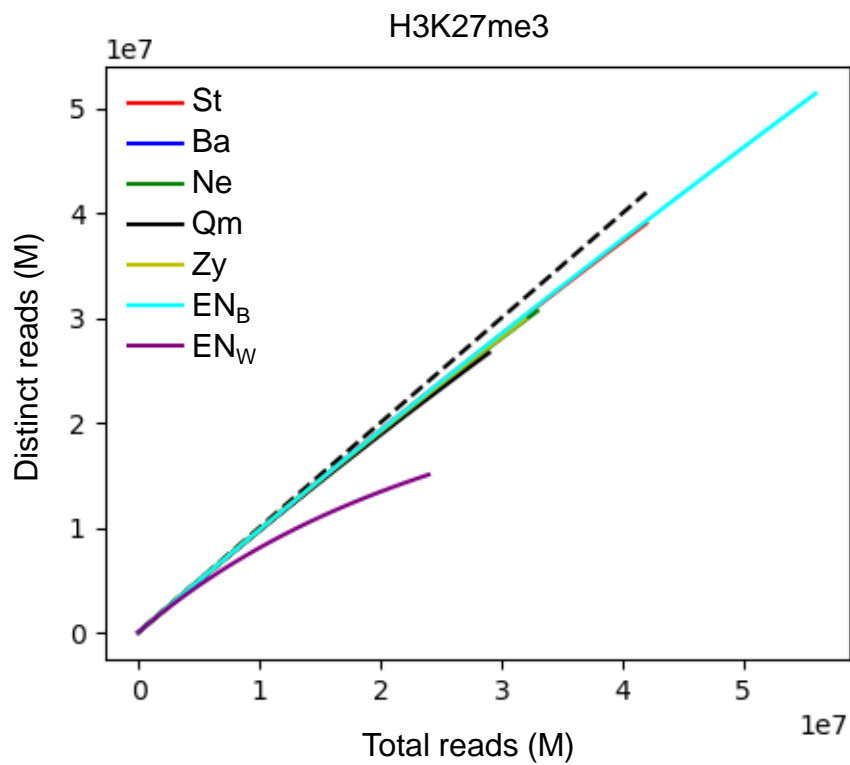

Supplement: Supplementary file 7 — Analysis of library complexity by Preseq package. The library complexity was estimated by Preseq package [8] for H3K4me3 and H3K27me3 ChIP-seq data from the ENCODE reference (ENB and ENw) and the corresponding datasets obtained either with standard protocol for the lab (St) or by using different purification reagents as identified in Fig. 1. (PDF 114 kb) [file 12864_2017_4371_MOESM7_ESM.pdf]

Additional file 9

A

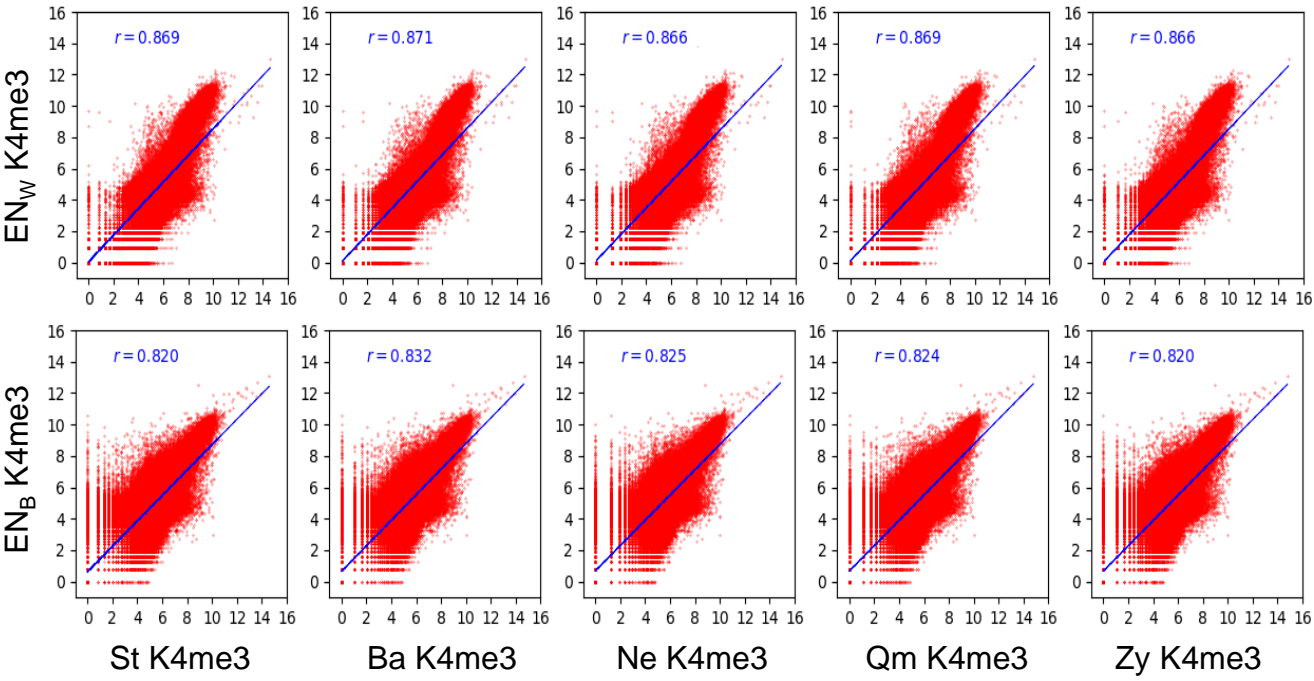

B

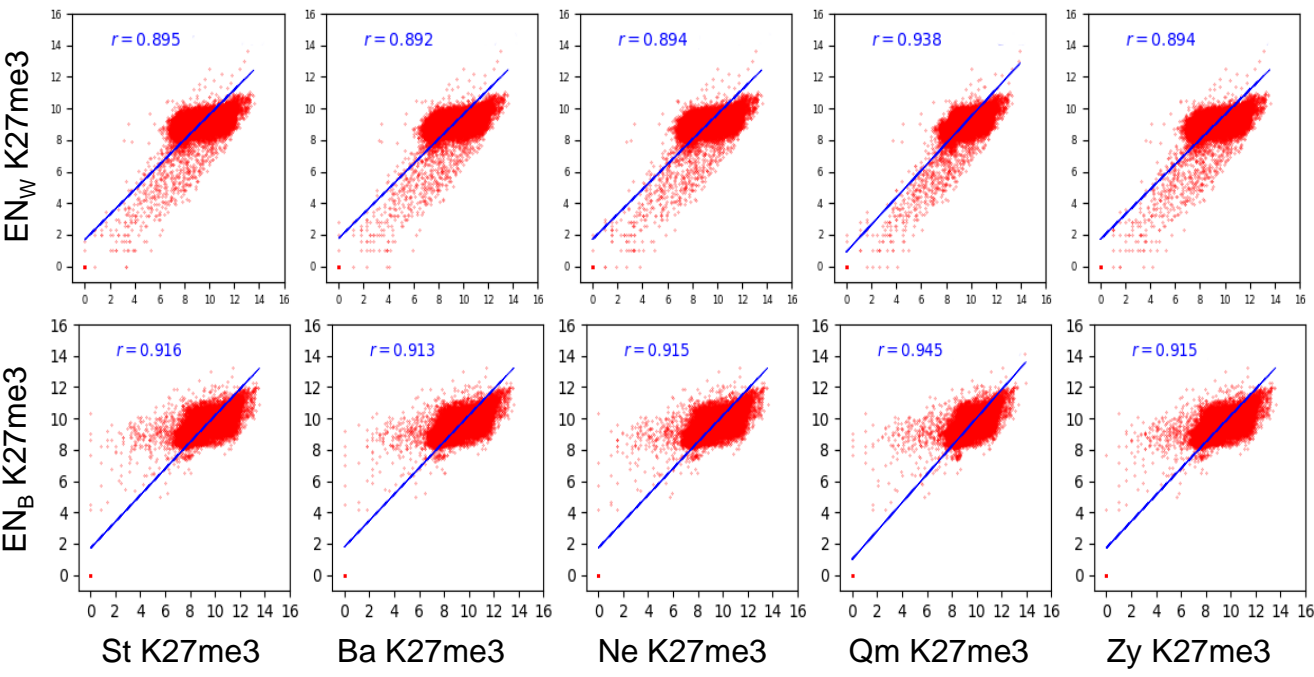

Supplement: Supplementary file 9 — ChIP-seq data generated with different purification reagents are highly correlated with the corresponding ENCODE datasets. Scatter plots showing the correlation between the ENCODE reference ChIP-seq data (ENB and ENw) and the corresponding datasets obtained either with standard protocol for the lab (St) or by using different purification reagents as identified in Fig. 1. The genome was divided into 5 kb bins for H3K4me3 and 100 kb bins for H3K27me3, and the number of mapped reads in the individual bins was calculated. r, Pearson correlation coefficient. P value in all correlation analysis was 0.001. A and B panels show H3K4me3 and H3K27me3 ChIP-seq data, respectively. (PDF 248 kb) [file 12864_2017_4371_MOESM9_ESM.pdf]

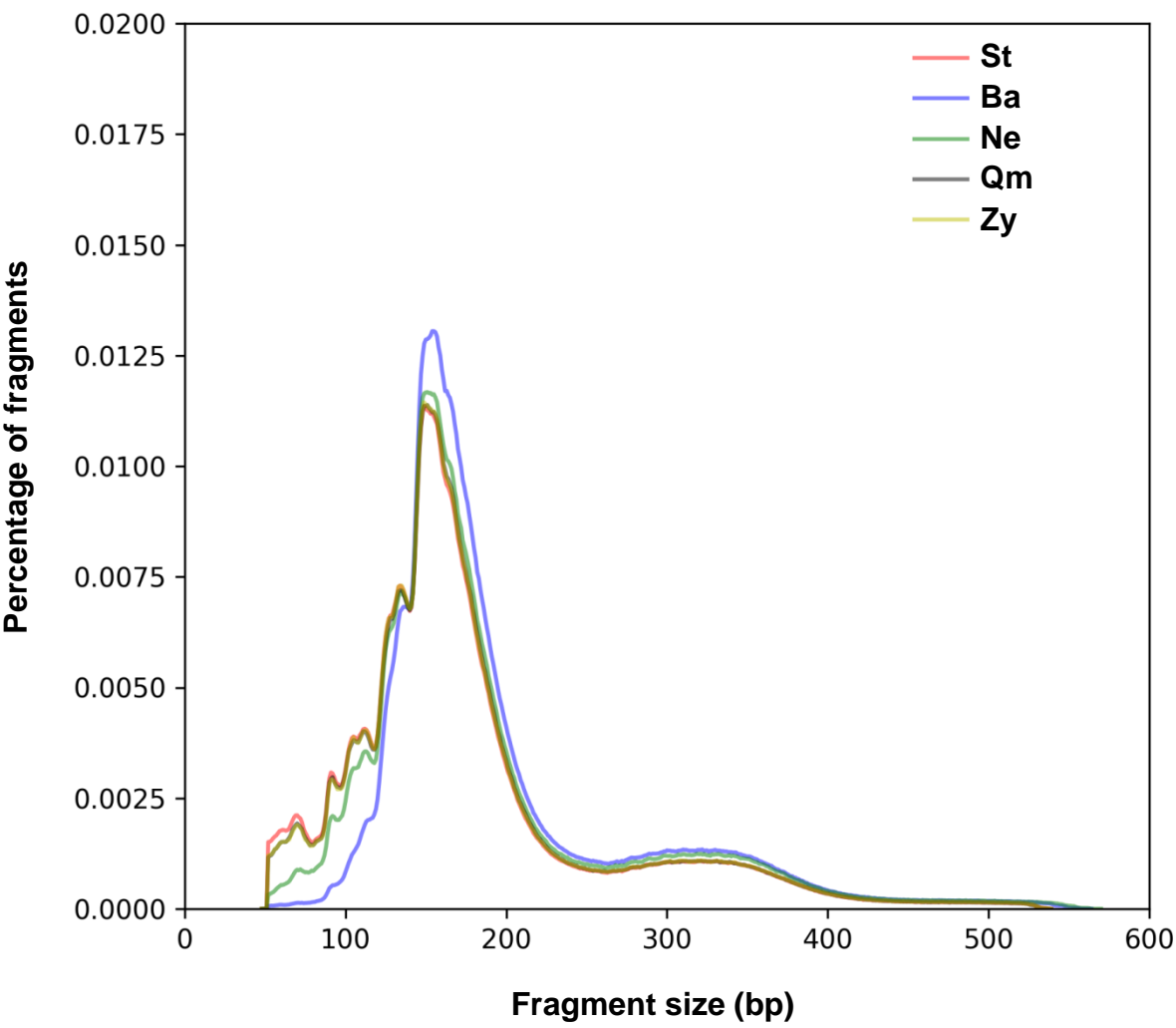

Supplement: Supplementary file 10 — Size distribution of sequencing reads from H3K4me3 ChIP-seq data. (PDF 203 kb) [file 12864_2017_4371_MOESM10_ESM.pdf]
