# Supplementary material for: Purification of nanogram-range immunoprecipitated DNA in ChIP-seq application
Source: BMC Genomics. 2017 Dec 21;18:985. doi: 10.1186/s12864-017-4371-5 (PMC5740926; doi:10.1186/s12864-017-4371-5)
Supplement: Supplementary file 2 — Microcentrifuge tubes tested in this study. (PDF 88 kb) [file 12864_2017_4371_MOESM2_ESM.pdf]

Additional file 2

Microcentrifuge tubes tested in this study

Tube name	Commercial supplier	Capacity	Material	Max RCF	Certifications/Compliance
MaxyClear Snaplock Microcentrifuge Tube	Axygen	1.7 mL	co-polymer /Polypropylene	14000 xg	Certified RNase-/DNase-free Nonpyrogenic
DNA LoBind Snap Cap PCR Tube	Eppendorf	1.5 mL	Polypropylene	30000 xg	DNA, DNase, RNase, PCR Inhibitors free
Siliconized Low-Retention Microcentrifuge Tube	Fisherbrand	1.5 mL	Polypropylene	30000 xg	Critical applications where minute sample recovery is important
Premium Microcentrifuge Tube	Fisherbrand	1.5 mL	Polypropylene	30000 xg	RNase, DNase-free
