# Supplementary material for: Purification of nanogram-range immunoprecipitated DNA in ChIP-seq application
Source: BMC Genomics. 2017 Dec 21;18:985. doi: 10.1186/s12864-017-4371-5 (PMC5740926; doi:10.1186/s12864-017-4371-5)
Supplement: Supplementary file 5 — Mapping results of ChIP-seq reads generated through different purification reagents. (PDF 94 kb) [file 12864_2017_4371_MOESM5_ESM.pdf]

# Additional file 5

**Mapping results of ChIP-seq reads generated through different purification reagents [7]**

| Library Name | Algorithm | Total # of Pairs | Total # Uniquely Mapped Pairs | Unique Pairs (%) | Complexity | Total Peaks | Duplicates (%) |
|---|---|---|---|---|---|---|---|
| Input | | 51,040,698 | 39,982,163 | 78.50% | 0.9349 | | 6.51% |
| St K4me3 | macs | 42,913,066 | 33,584,141 | 78.50% | 0.9251 | 37,679 | 7.49% |
| Qm K4me3 | macs | 28,610,640 | 22,443,663 | 78.70% | 0.9158 | 34,228 | 8.42% |
| Ne K4me3 | macs | 24,329,553 | 19,232,263 | 79.30% | 0.8745 | 34,498 | 12.55% |
| Zy K4me3 | macs | 28,444,136 | 22,232,194 | 78.40% | 0.9330 | 34,923 | 6.70% |
| Ba K4me3 | macs | 29,818,138 | 23,595,325 | 79.40% | 0.9238 | 35,282 | 7.62% |
| St K27me3 | sicer | 41,332,165 | 33,133,641 | 80.30% | 0.9369 | 30,323 | 6.31% |
| Qm K27me3 | sicer | 30,060,890 | 23,524,187 | 78.40% | 0.9248 | 33,513 | 7.52% |
| Ne K27me3 | sicer | 33,067,413 | 26,534,649 | 80.40% | 0.9340 | 26,714 | 6.60% |
| Zy K27me3 | sicer | 31,501,790 | 25,243,738 | 80.30% | 0.9407 | 29,099 | 5.93% |
| Ba K27me3 | sicer | 25,693,025 | 20,581,001 | 80.30% | 0.9370 | 40,895 | 6.30% |
