# Supplementary material for: Purification of nanogram-range immunoprecipitated DNA in ChIP-seq application
Source: BMC Genomics. 2017 Dec 21;18:985. doi: 10.1186/s12864-017-4371-5 (PMC5740926; doi:10.1186/s12864-017-4371-5)
Supplement: Supplementary file 8 — Overlapping percentage of peaks relative to ENCODE data. (PDF 91 kb) [file 12864_2017_4371_MOESM8_ESM.pdf]

Additional file 8

Overlapping percentage of peaks relative to ENCODE data

Library Name	Total Peaks	Overlap	Un-overlap	% Overlap	% Un-overlap
St K4me3	37,679	22,471	15,208	59.64%	40.36%
Qm K4me3	34,228	22,130	12,098	64.65%	35.35%
Ne K4me3	34,498	22,546	11,952	65.35%	34.65%
Zy K4me3	34,923	22,628	12,295	64.79%	35.21%
Ba K4me3	35,282	22,238	13,044	63.03%	36.97%
St K27me3	30,323	20,910	9,413	68.96%	31.04%
Qm K27me3	33,513	24,414	9,099	72.85%	27.15%
Ne K27me3	26,714	18,804	7,910	70.39%	29.61%
Zy K27me3	29,099	20,415	8,684	70.16%	29.84%
Ba K27me3	40,895	28,626	12,269	70.00%	30.00%

The ENCODE data from University of Washington was used for H3K4me3.

The ENCODE data from Broad institute was used for H3K27me3.
