# Supplementary material for: Purification of nanogram-range immunoprecipitated DNA in ChIP-seq application
Source: BMC Genomics. 2017 Dec 21;18:985. doi: 10.1186/s12864-017-4371-5 (PMC5740926; doi:10.1186/s12864-017-4371-5)
Supplement: Supplementary file 11 — Purification regents tested in this study. (PDF 153 kb) [file 12864_2017_4371_MOESM11_ESM.pdf]

Additional file 11

Purification reagents tested in this study

Kit name	Commercial supplier	Binding capacity	DNA size range	Elution volume	Features
ChIP DNA Clean & Concentrator	Zymo	Up to 5 µg	50 bp to ~ 23 kb	6-100 µl	Silica-based column
Wizard® SV Gel and PCR Clean-Up	Promega	10 ng to 40 µg	100 bp to 10 kb	15-50 µl	Silica-based column
GeneJET PCR Purification Kit	Thermo Fisher	Up to 25 µg	25 bp to 20 kb	10-50 µl	Silica-based column
PureLink® PCR Purification Kit	Invitrogen	Up to 40 µg	100 bp to 12 kb	50 µl	Silica-based column
Monarch® PCR & DNA Cleanup Kit	New England Biolabs	Up to 5 µg	~50 bp to 25 kb	6–20 µl	Silica-based column
Chromatin IP DNA Purification Kit	Active Motif	100 ng to 1 µg	≥~50 bp	10-150 µl	Silica-based column
QIAquick PCR Purification Kit	Qiagen	10 µg	100 bp to 10 kb	> 30 µl	Silica-based column
MinElute PCR Purification Kit	Qiagen	5 µg	70 bp – 4 kb	> 10 µl	Silica-based column
Agencourt AMPure XP	Beckman	NA	≥~100 bp	> 15 µl	SPRI-based beads
Agencourt RNAClean™ XP	Beckman	NA	NA	> 15 µl	SPRI-based beads

NA: not available
